# Neuronal Correlates of Small Animal Phobia in Human Subjects through fMRI: The Role of the Number and Proximity of Stimuli

**DOI:** 10.3390/life11040275

**Published:** 2021-03-26

**Authors:** Ascensión Fumero, Rosario J. Marrero, Francisco Rivero, Yolanda Alvarez-Pérez, Juan Manuel Bethencourt, Manuel González, Wenceslao Peñate

**Affiliations:** 1 Departamento de Psicología Clínica, Psicobiología y Metodología, Facultad de Psicología, Universidad de La Laguna, 38200 La Laguna, Tenerife, Spain; rmarrero@ull.edu.es (R.J.M.); friverop@ull.edu.es (F.R.); jmbethen@ull.edu.es (J.M.B.); mgonzaro@ull.edu.es (M.G.); wpenate@ull.es (W.P.); 2 Facultad de Ciencias de la Salud, Universidad Europea de Canarias, 38300 La Orotava, Tenerife, Spain; 3 Servicio Canario de la Salud, 38004 Santa Cruz de, Tenerife, Spain; yolanda.alvarezperez@sescs.es

**Keywords:** small-animal phobia, fMRI, number, proximity, virtual reality, video images

## Abstract

Brain regions involved in small-animal phobia include subcortical and cortical areas. The present study explored the neuronal correlates of small-animal phobia through fMRI data to determine whether a manipulation of number and proximity parameters affects the neurobiology of the processing of feared stimuli. The participants were 40 individuals with phobia and 40 individuals without phobia (28.7% male and 71.3% female). They watched videos of real and virtual images of spiders, cockroaches and lizards in motion presented more or less nearby with one or three stimuli in the different conditions. The results suggested a differential brain activity between participants with and without phobia depending on the proximity and number of phobic stimuli. Proximity activated the motor response marked by the precentral gyrus and the cingulate gyrus. By contrast, the number of stimuli was associated with significant sensory activity in the postcentral gyrus and ventromedial prefrontal cortex. We also observed a greater activity in the occipital cortex when exploring the number compared to the proximity factor. Threatening stimuli presented nearby and those presented in greater numbers generated an intense phobic response, suggesting a different emotion regulation strategy. Based on these findings, exposure therapies might consider including proximity to the threat and number of stimuli as key factors in treatment.

## 1. Introduction

Small-animal phobia is a type of specific phobia that produces an exaggerated and unreasonable fear in individuals when faced with the feared stimuli. It is considered as an anxiety- and fear-related disorder [1]. Several studies have focused on identifying the neurobiology of the processing of feared stimuli [2,3]. The amygdala and insular cortex form the core of a neurobiological anxiety network that is common to specific anxiety disorders [4,5]. The fMRI procedure has been tested as a gold standard for in vivo imaging of human brain activity in response to various sensory stimuli [6]. Considering this, the goal of this research was to use fMRI to identify the brain regions associated with small-animal phobia in healthy and phobic individuals and explore the role of specific properties of the stimuli, such as the proximity to the phobic stimuli and the number of small animals presented.

Two pathways can be distinguished: a fast and shorter pathway triggered by acute fear and a slower and longer pathway involved in the acquisition and anticipation of the threat [7,8]. Thus, the fear response has a subjective component [9] and a physiological component that has been studied through the predatory imminence theory [7,10,11]. According to this theory, defensive behavior depends on the threatening stimulus or predatory imminence, which is in turn influenced by the physical and temporal distance from the stimulus. Some studies have found that when the threatening stimulus is close, there is a change in brain activity from prefrontal cortical areas to midbrain areas, such as the mid-dorsal anterior cingulate cortex [12].

The neural mechanisms involved in fear inhibition usually overlap with those involved in emotional regulation, which is associated with prefrontal-limbic connectivity and involves various brain regions, such as the ventromedial prefrontal cortex (vmPFC), dorsolateral prefrontal cortex (DLPFC), anterior cingulate cortex (ACC), orbitofrontal cortex (OFC) and right amygdala [13]. A greater connectivity of the right amygdala with the ventromedial prefrontal cortex during the presentation of aversive pictures has been observed in healthy participants using real-time fMRI neurofeedback [14]. Several studies have identified brain regions that are activated in response to phobic stimuli. The OFC, the middle temporal gyrus and the insula seem to be associated with the presentation of feared stimuli [15]. Moreover, studies have reported activation in the left insula, left amygdala, right thalamus and cerebellum in phobic participants compared to healthy controls [16]. These studies have found that the same brain structures associated with feared stimuli (i.e., the amygdala, anterior insula and ACC) are involved in the regulation of emotions of healthy individuals [17].

There is a debate about which properties or characteristics of phobic stimuli are necessary to trigger an anxiety response in the brain. Data suggest that virtual reality phobic stimuli with immersive properties (i.e., sense of presence) can activate the anxiety neural circuitry in a similar way to real phobic stimuli [18]. It has been questioned how other characteristics, such as the proximity and clarity of the phobic stimulus, affect the perception of the threatening stimulus. There is evidence that stimuli that emit affective signals of threat are perceived as being physically closer than those that do not emit affective signals [19]. Moreover, it has been observed that the relative proximity of an animal in images with greater (i.e., head-centered) or lesser (i.e., whole body) clarity affects its rapid detection [20]. Particularly, behavioral studies have found that rapid fear detection relies on higher clarity rather than lower clarity [21]. This finding is indicative of an involvement of cortical visual areas that supports the view that the cerebral cortex is crucial for the processing of ecologically relevant signals. This view challenges the idea that a subcortical pathway to the amygdala is essential for the initial processing of fear signals. It can be speculated that phobic stimuli imaging may require less attention in phobic individuals than in non-phobic controls because of the greater ability of the former to identify the threat induced by a rapid activation of the amygdala instead of visual areas [22]. However, other opposing results demonstrated that the speed of detection of the phobic stimulus was greater with whole-body snake images than with head-centered images [20]. Specifically, it was found that the perceived proximity of the feared stimulus activated the dorsal anterior cingulate, the mid-insula and the ventrolateral prefrontal cortex in females with spider phobia compared to healthy participants [23]. Nevertheless, the activation of the amygdala in threat monitoring was observed in both phobic and healthy participant groups. In general, it seems that the spatial distance to the stimulus can influence the type of response and the brain areas involved but that it is also necessary to consider how the individual perceives that distance. Apart from physical aspects, certain psychological aspects such as a greater number of stimuli may influence the fear response. To our knowledge, however, no previous studies have explored whether the number of feared stimuli generates a greater activation in different brain areas. Self-reported fear may be associated with the number of stimuli and thus be another factor involved in the psychological response [7].

In this study, we analyzed the neuronal correlates of small-animal phobia to determine whether a manipulation of the number and proximity parameters affects the neurobiology of the processing of feared stimuli. Specifically, we explored whether the proximity and number of phobic stimuli are associated with an increased anxiety level when virtual reality and real phobic stimuli are presented. In addition, we analyzed the different brain regions activated, comparing participants with small-animal phobia and non-phobic control participants. In line with previous studies, we hypothesized that stimuli that were presented closer would be perceived as more threatening and would activate the same brain regions (i.e., the amygdala, thalamus, visual regions and ventromedial prefrontal cortex) in phobic and non-phobic participants. This hypothesis was developed considering that there are other brain regions that are mostly activated in phobic participants, namely the dorsal anterior cingulate, the medial insula and the ventrolateral prefrontal cortex.

## 2. Materials and Methods

### 2.1. Participants

The sample consisted of 80 adults (28.7% male and 71.3% female) who lived in Tenerife (Canary Islands, Spain). They were assigned to one of two groups: 40 were diagnosed with small-animal phobia (20% males, mean age = 34.05 years, SD = 11.03) and 40 were non-phobic controls (37.5% males, mean age = 21.65 years, SD = 4.53).

All participants were right-handed and none of them had any visual problems. The inclusion criteria were the following: being an adult with a diagnosis of specific phobia according to the scores in questionnaires on specific phobia and anxiety and through a clinical interview; the phobia had to be the primary psychological disorder and not be explained by another health condition; participants should not be receiving any treatment for specific phobia at the time of the study and not show any impediment to undergoing a magnetic resonance imaging session.

### 2.2. Instruments

The Composite International Diagnostic Interview (CIDI), Version 2.1 [24] was used to verify the diagnosis of phobia. The CIDI is a structured interview for major mental disorders according to the CIE-10 criteria [25]. For the purposes of this study, questions related to specific phobia, social phobia, agoraphobia and panic attacks were selected. Participants diagnosed with specific small-animal phobia were included (F40.218).

The S-R Inventory of Anxiousness [26] is a 14-item inventory with a 5-point Likert-type scale ranging from 0 to 3 that assesses physiological, cognitive and behavioral anxiety symptoms associated with an anxiety-inducing situation. The phobic stimulus target is pointed out prior to the participant’s response. The inventory has shown high internal consistency (0.95) and adequate convergent validity [26,27]. For the current study, Cronbach’s alpha was 0.79.

The Hamilton Anxiety Rating Scale (HARS) [28] is a 14-item clinician administered scale with a 5-point Likert-type scale ranging from 0 (not present) to 4 (very severe) that assesses the severity of each anxiety symptom. The scale has a high internal consistency of 0.92 [29] and a good test–retest reliability of 0.86 [30]. For the current study, Cronbach’s alpha was 0.72.

Hand preference was assessed with the Edinburgh Handedness Inventory (EHI) [31]. The EHI consists of ten items: writing, drawing, throwing, using scissors, toothbrush, knife (without fork), spoon, broom, striking a match, and opening a box. Participants indicated the strength of their hand preference for each of the 10 items by putting two or one tick in the appropriate column, or one tick in each column if they were indifferent about that item. The EHI provides a Laterality Quotient ranging from +100 (totally right-handed) to −100 (totally left-handed).

### 2.3. Design

We performed a 3T GE fMRI study to compare three primary measures with two levels each: video image (real images vs. virtual reality), proximity (near vs. far) and number of phobic stimuli (one vs. several). Participants were presented with a 20 s block presentation of videos with spiders, cockroaches and lizards. Stimuli were recorded in 3D and projected in the MRI scanner in stereoscopic 3D video using Visual Stim digital MRI-compatible 3D glasses (graphics card: GeForce 8600GT). Each participant was randomly presented with 16 blocks of phobic images.

The stimuli consisted of small animals in motion. The presentation modality effects were controlled using 3D recorded movies as the models to create the virtual reality stimuli. In the initial fMRI session, the fear-arousing properties of the virtual reality stimuli were tested by observing brain activation. Stimulus valence was not assessed because the stimuli were directly related with each specific phobia. Participants were randomly assigned to one of two groups: one received the stimuli in virtual reality format (VR group) and the other received them as real images (RI group).

**Figure 1:**
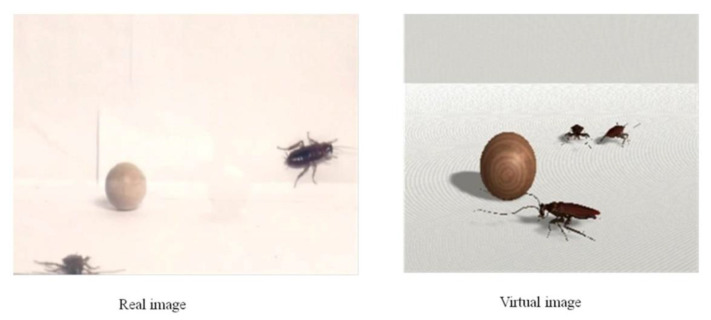
Examples of the real image (RI) and virtual reality (VR) stimuli.

Wooden balls were added to the images of the phobic stimuli so that participants could observe the depth or three-dimensional effect with greater quality. Examples of the real image (RI) and virtual reality (VR) stimuli are presented in Figure 1.

The number variable was created by presenting a single image or three images of the corresponding animal together. The proximity variable was manipulated by presenting videos with focal lengths close to 25 mm—considered distant—or close to 65 mm—considered nearby. Whole-brain voxel-wise analyses were performed.

### 2.4. Procedure

The study took place from April to July 2020. Phobic participants were recruited through various media (i.e., website, press, flyers, radio, TV and newspapers). Later, an e-mail with the inventories was sent to possible participants. The initial diagnosis of specific phobia according to participants’ inventory scores was corroborated by a semi-structured interview. Those who did not meet the inclusion criteria were excluded. Participants signed an informed consent form before the start of experiment, which was approved by the Ethics Committee for Research and Animal Welfare of the University of La Laguna (ref. CEIBA2012-0033). Participants were exposed to real and virtual video images of small animals (i.e., cockroaches, spiders or lizards). The phobic stimuli matched each individual’s specific phobia. After their participation, subjects were entitled to receive an eight-session free psychological treatment for specific animal phobia in exchange for their participation.

### 2.5. fMRI Data Acquisition

Functional MRI data were collected with a 3T General Electric Signa Excite scan. The BOLD signal was measured with an echo planar imaging sequence with 30 ms of echo time, 2000 ms repetition time, 25.6° field of view and 75° flip angle. The image dimension was 64 × 64 × 32 mm with 4 × 4 × 4 mm voxel dimension.

### 2.6. fMRI and Data Analysis

Brain images were analyzed with Statistical Parametric Mapping (SPM12) software [32]. Pre-processing procedures included realigning, co-registering, segmenting (with forward deformation fields), normalizing (structural images with a 1 × 1 × 1 mm voxel size and functional images with a 4 × 4 × 4 mm voxel size) and smoothing (Gaussian Kernel of 8 mm, FWHM). Images were rendered and adjusted to the standard brain template of the Montreal Neurological Institute (MNI). Motion correction was applied to preprocessing of functional magnetic resonance imaging (fMRI) data to maximize sensitivity and minimize false activations. When a motion was detected by a motion alarm, the participant was eliminated. Next, data in which a 2 mm frame-to-frame head movement was detected were also removed. Additionally, a high-resolution T1-weighted anatomical volume was recorded. The FSPGR 3D protocol used ASSET for General Electric Signa Excite HD 3T with TR: 8852 msec, TE: 1756 msec, FA: 10°. The image dimension was 256 × 256 × 172 mm and the voxel dimension was 1 × 1 × 1 mm with FOV: 25.6 mm^2^ and TI: 650 msec.

Previously, an ANOVA of anxiety measures between both groups with and without phobia was performed to test internal validity. The 2 × 2 × 2 factor design was tested with a three-way ANOVA to compare the main effects of image format, proximity and number of stimuli and the interaction effect between them on whole-brain activation. The regions were extracted from the WFU Pickatlas 3.0.5b [33] for SPM12 with the Automated Anatomical Labeling (AAL2) brain atlas [34].

The Family-Wise Error (*p* < 0.05 FWE corrected) correction was used. However, non-corrected probabilities were admitted when they were congruent with the biological model of phobias (but never higher than 0.001 uncorrected). The error was corrected considering that there was activation when the activated area was equal to or greater than a 3-voxel cluster (k) with a voxel size of 4 × 4 × 4 mm. When the uncorrected criterion of *p* < 0.001 and k > 3 was 192 mm^3^, activation was considered.

## 3. Results

We found significant differences between participants with and without phobia in anxiety measures. Participants with phobia scored higher in S-R (F(1,78) = 624.04, *p* < 0.001)) and HARS (F(1,78) = 110.95, *p* < 0.001)) measures. No significant sex or age differences were found in the S-R questionnaire. However, in the non-phobic group, males had slightly higher anxiety scores than females, while in the phobic group, females had higher scores than males (HARS: (F(1,79) = 8.86, *p* < 0.01)). Moreover, in the group with phobia, older participants showed greater anxiety than younger ones (HARS: (F(1,79) = 10.83, *p* < 0.01)). No significant differences were found between the VR and RI format in anxiety (S-R: (F(1,78) = 0.02, *p* = 0.896)); VR group M = 19.30, SD = 19.97; RI group M = 18.72, SD = 19.30; HARS: (F(1,78) = 1.05, *p* = 0.309)); VR group M = 12.18, SD = 12.88; RI group M = 9.55, SD = 9.82).

A three-way ANOVA (whole-brain analysis) was performed with video image format (virtual vs. real), proximity of stimuli (near vs. far) and number of phobic stimuli (one vs. three) as independent variables for each group separately. The whole-brain activations are shown in Table 1. The value considered as the F-score threshold was 11.26. The threshold was determined according to FWE-corrected values (*p* < 0.05) and uncorrected values (*p* < 0.001 and k > 3). SPM12 provides an F value to avoid false positives. Next, it calculates significant activations with the F statistic and corresponding *p*.

In participants with phobia, the video image format, proximity and number interaction effect was not significant. However, a proximity by number interaction effect was statistically significant (F(1,152) = 11.73, *p* < 0.001)). There was greater brain activation in the vermis when stimuli were presented nearby and in greater numbers. The video image format by proximity interaction effect (F(1,152) = 12.70, *p* < 0.001)) was also significant. Medial cingulate activation was greater with real video images presented nearby.

**Figure 2:**
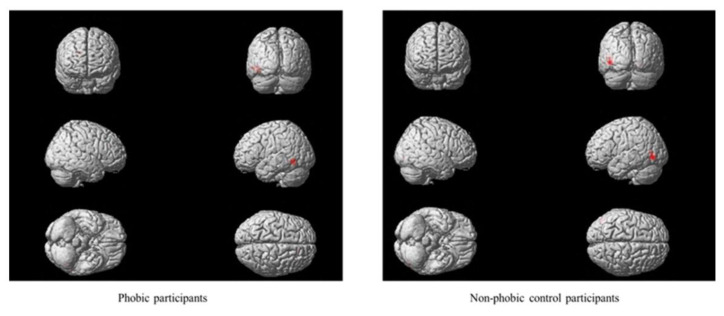
Main effects of image format.

**Figure 3:**
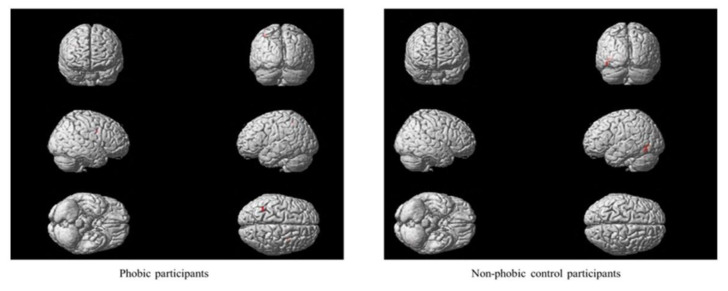
Main effects of proximity.

**Figure 4:**
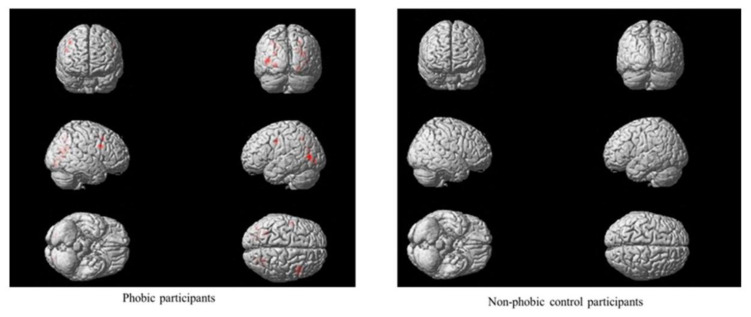
Main effects of number.

Main effects were found in participants with phobia regarding the video image format, proximity and number of stimuli. Significant differences between real and virtual video images were observed in two brain region activations: the left inferior temporal and the right anterior cingulate gyri (see Figure 2). Moreover, feared stimuli presented nearby generated higher brain activation than those presented from a greater distance in brain regions such as the vermis, right precentral gyrus, right medial cingulate gyrus and left inferior parietal gyrus (see Figure 3). Additionally, a greater number of stimuli activated several brain regions: the right medial and inferior opercularis prefrontal gyrus, left inferior parietal gyrus, left postcentral gyrus, lingual and angular gyrus, left cuneus, right calcarine region, bilateral superior and medial occipital gyrus, and left inferior occipital gyrus (see Figure 4).

In the group of participants without phobia, the video image format, proximity and number interaction effect were significant (F(1,152) = 13.04, *p* < 0.001). Main effects of the video image format and proximity were found (see Figure 2 and Figure 3). Significant differences between real and virtual video images were found on the left side of the inferior occipital, medial temporal, right lingual gyri and vermis. Besides, nearby stimuli generated higher brain activation than stimuli presented from a distance on the left side of the medial occipital gyrus and calcarine region and the right medial and left inferior temporal brain regions.

## 4. Discussion

In this research, the effect of proximity and the number of feared stimuli presented in real and virtual video images to participants with and without small-animal phobia were analyzed. fMRI was used to identify which brain regions were involved in threat monitoring and determine whether different areas were activated depending on the proximity and number of stimuli. The results showed a greater intensity of activation and a greater number of structures involved in participants with phobia compared to participants without phobia. Video image format activated similar brain fear processing circuits in participants with phobias. Proximity activated the motor response marked by the precentral and cingulate gyri. The number of feared stimuli activated sensory areas, mainly in the postcentral gyrus and ventromedial prefrontal cortex.

Comparisons based on the presentation of the images and the number and proximity of feared stimuli in the group of participants with phobia revealed that different areas involved in emotional regulation were activated. Specifically, the video image format activated the inferior temporal brain region, since the task involved visual recognition, and the anterior cingulate cortex associated to the processing of conflictive situations and emotional regulation. Activation of the cingulate cortex was both specific to the clinical group and phobic stimuli [16]. The cingulate is part of an attentional network encompassing the prefrontal cortex, parietal cortex and supplementary motor areas and facilitates the assessment of emotional salience, allowing for regulation of the subsequent emotional response to feared stimuli. The proximity of the feared stimulus activated higher-order sensory association areas including the parietal cortex and medial cingulate, which are implicated in decision making associated with motor behaviors.

Similarly, the vermis of the cerebellum, which is associated with the planning and initiation of movement (i.e., escape behavior), was affected by proximity of the feared stimulus in participants with phobia. Previous neuroimaging studies have highlighted increased cerebellar changes [16,35,36] associated with specific phobia and shown the relevance of the cerebellum as a potential clinical marker of anxiety [37] related to difficulties in emotion regulation [38]. The number of feared stimuli was associated with greater activation of different brain areas related mainly to a larger network of the motor system, including the precentral cortex and attentional network. Findings on the greater activation of multiple motor regions in participants with phobia may imply a more intense fight-or-flight response [39]. These results suggest that phobic responses activate the same brain regions identified in fear but the activation is stronger and involves larger brain activity networks.

In the non-phobic control group, the vermis was more activated with certain image formats than others. Previous results have found that the presentation of stimuli in virtual reality facilitates the response to proximal threatening stimuli [40]. The format in which visual information is presented may make a difference in neural circuits activated during the detection of nearby threats. An activation of visual areas in the non-phobic control group was identified according to video image format and the proximity of the feared stimulus. The activation occipital regions in the presence of threatening stimuli suggests high visual processing and vigilance that is frequently associated with fear [39]. A meta-analysis showed similar results in the control group and also in regions of the temporal and parietal cortex [16]. Previous research has found that the thalamus and visual regions were modulated by levels of subjective anxiety in healthy controls and participants with spider phobia, while the dorsal anterior cingulate and insula were activated in individuals with spider phobia [41,42].

This study has several limitations. First, the small sample size and age differences may have affected the robustness of its results. Second, the effect of sex was not evaluated because few males reported small-animal phobia. Previous studies show that sex plays an important role in modulating responses related to phobia. Females show higher specific phobia lifetime prevalence compared to males [43]. Additionally, different neural mechanisms related to emotion processing between males and females have been found [44]. Third, considering that SPM software for analyzing the interaction between numerous variables with several levels is limited, between-group comparations were not possible. Fourth, this study only used small-animal phobia without considering other possible comorbid phobias. Fifth, participants’ level of disgust was not assessed as an emotional state different from fear/phobia; their escape behavior was not assessed either. Sixth, real phobic stimuli were filmed in 3D as a representative condition to achieve in vivo exposure but this equivalence was not tested. Finally, proximity and number measures need to be validated beforehand because the minimum level of stimulation that can generate an intense phobic response has not been determined. In the future, it would be interesting to analyze whether a full image of the animal or an image of specific parts of the animal is more threatening and is reflected in the activation of specific brain areas.

This experimental design made it possible to identify the different brain regions involved in an exaggerated fear response by manipulating the proximity and number parameters. Brain regions such as the cingulate cortex, occipital, inferior parietal and mid-frontal areas were associated with small-animal phobia when feared stimuli were presented nearby and in greater numbers. Identifying the mechanisms involved in fear is essential to design an effective treatment for phobias. Anxiety disorders imply a greater perception of imminent threat so it would be advisable for exposure therapies to include proximity to the threat as a key factor in treatment [7].

## Figures and Tables

**Table 1:** Brain areas activated by image, proximity and number in phobic participants and non-phobic control participants.

AREA	Coordinates	Hemisphere	K	Z	F	*p*
**Participants with Phobia**						
**Image**						
Inferior temporal	−42, −60, −6	Left	26	4.03	18.65	0.0000
Anterior cingulate	18, 32, 26	Right	6	3.60	14.99	0.0002
**Proximity**						
Vermis	6, −48, −10		2	3.56	14.71	0.0002
Precentral	34, 4, 34	Right	6	3.40	13.49	0.0003
Medial cingulate	18, 0, 42	Right	3	3.29	12.66	0.0005
Inferior parietal	−38, −52, 54	Left	3	3.19	11.97	0.0007
**Number**						
Medial occipital	−42, −72, 6	Left	25	4.92	27.93	0.0000
Medial occipital	42, −68, 6	Right	5	3.51	14.29	0.0002
Inferior occipital	−26, −84, −6	Left	14	3.89	17.41	0.0001
Superior occipital	26, −64, 42	Right	13	3.76	16.30	0.0001
Superior occipital	−26, −72, 18	Left	18	3.68	15.69	0.0001
Inferior frontal opercularis	50, 12, 26	Right	18	3.74	16.15	0.0001
Medial frontal	42, 20, 42	Right	7	3.61	15.11	0.0002
Inferior parietal	−30, −56, 42	Left	17	3.77	16.44	0.0001
Postcentral	−54, −4, 42	Left	8	3.74	16.17	0.0001
Lingual	22, −88, −10	Right	9	4.26	20.83	0.0000
Angular	34, −60, 22	Right	38	3.65	15.37	0.0001
Calcarine	30, −76, 6	Right	7	3.48	14.05	0.0003
Cuneus	−18, −76, 34	Left	5	3.41	13.56	0.0003
**Participants without Phobia**						
**Image**						
Inferior occipital	−46, −76, −6	Left	22	4.70	25.46	0.0000
Medial temporal	−46, −68, 6	Left		3.76	16.34	0.0001
Lingual	18, −88, −6	Right	3	3.50	14.24	0.0002
Vermis	6, −36, −6		3	3.36	13.19	0.0004
**Proximity**						
Medial occipital	−38, −68, −2	Left	4	3.47	14.00	0.0003
Calcarine	−22, −64, 18	Left	2	3.44	13.77	0.0003
Medial temporal	42, −68, −2	Right	2	3.22	12.13	0.0006
Medial temporal	−46, −68, −10	Left	3	3.20	12.00	0.0007
**Number**						
not significant						
